# Exploring monkeypox virus proteins and rapid detection techniques

**DOI:** 10.3389/fcimb.2024.1414224

**Published:** 2024-05-28

**Authors:** Kamila Sagdat, Assel Batyrkhan, Damira Kanayeva

**Affiliations:** Department of Biology, School of Sciences and Humanities, Nazarbayev University, Astana, Kazakhstan

**Keywords:** monkeypox virus, mpox, extracellular enveloped virus proteins, intracellular mature virus proteins, profilin-like proteins, detection, biosensor

## Abstract

Monkeypox (mpox) is an infectious disease caused by the mpox virus and can potentially lead to fatal outcomes. It resembles infections caused by viruses from other families, challenging identification. The pathogenesis, transmission, and clinical manifestations of mpox and other *Orthopoxvirus* species are similar due to their closely related genetic material. This review provides a comprehensive discussion of the roles of various proteins, including extracellular enveloped virus (EEV), intracellular mature virus (IMV), and profilin-like proteins of mpox. It also highlights recent diagnostic techniques based on these proteins to detect this infection rapidly.

## Graphical Abstract

GRAPHICAL ABSTRACT
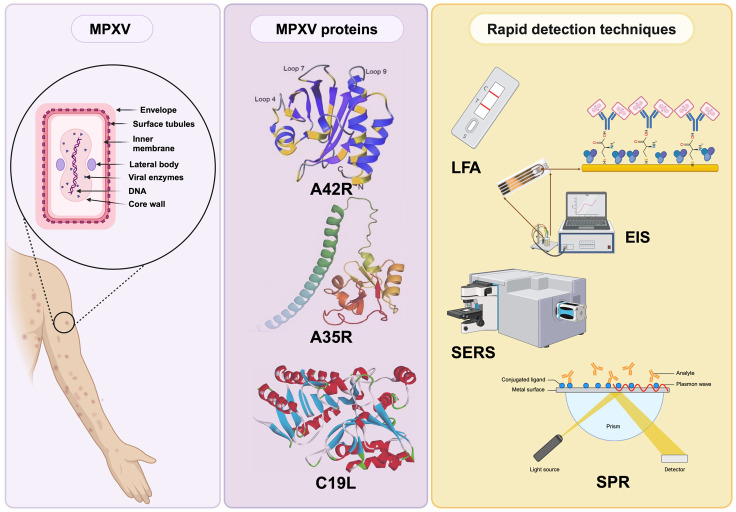

## Introduction

Mpox (formerly referred to as monkeypox) is a zoonotic infectious disease caused by the monkeypox virus (MPXV) that belongs to the *Orthopoxvirus* (OPXV) genus of the *Poxviridae* family (Gubser et al., 2004). It results in symptoms that start with fever, headache, and back pain and are followed by systematic rash and blisters (Magnus et al., 1959; Cho and Wenner, 1973). These symptoms are similar to infections caused by other members of the same genus, such as the variola virus (VARV) and vaccinia virus (VACV). In addition, mpox resembles infections caused by viruses from other families. For example, chickenpox, caused by the varicella-zoster virus, a member of the *Herpesvirus* family, exhibits symptoms similar to MPXV infections (Chauhan et al., 2023). Since the clinical presentation of these diseases in humans shares similarities, diagnosing mpox relying on the observable symptoms is challenging.

Mpox has the potential to lead to a fatal outcome, resulting in a mortality rate ranging from 1% to 10%, depending on the specific clade of the MPXV strain causing the infection and the level of access to advanced healthcare services (Adalja and Inglesby, 2022). At present, no specific treatment is available for the MPXV infection. However, supportive care plays a vital role in managing the symptoms, including using medications to alleviate fever and pain. The smallpox vaccine can provide partial protection (85%) against monkeypox, but its effectiveness is not guaranteed in all cases (Fine et al., 1988). Hughes et al. (2014) discovered a specific epitope of the MPXV through a monoclonal antibody designed for the heparan binding site on the MPXV envelope protein. Hence, this finding paved the way for more specific serologic assays for mpox detection (Al-Musa et al., 2022).

Current MPXV detection methods include viral isolation, electron microscopy, immunohistochemistry, and PCR (polymerase chain reaction)/rtPCR (reverse transcription PCR) (Li et al., 2006; McCollum and Damon, 2014; Karagoz et al., 2023). However, these techniques require advanced technical skills, state-of-the-art laboratories, and specialized training and fail to meet the demand for timely and rapid identification of MPXV infection (McCollum and Damon, 2014; Halvaei et al., 2023). Consequently, there is a need for reliable detection approaches to accurately identify MPXV-infected individuals and control the spread of the disease. In this paper, our focus centers on the pathogenesis and biological traits of mpox while also detailing the characterization of MPXV envelope proteins, including A29L, H3L, E8L, M1R, L1R, C19L, A35R, B6R, and the profilin-like mpox A42R protein. These proteins are highlighted as potential targets for a range of detection methods. Additionally, we provide an overview of recent advancements in rapid detection techniques for mpox.

## Epidemiology

**Figure 1:**
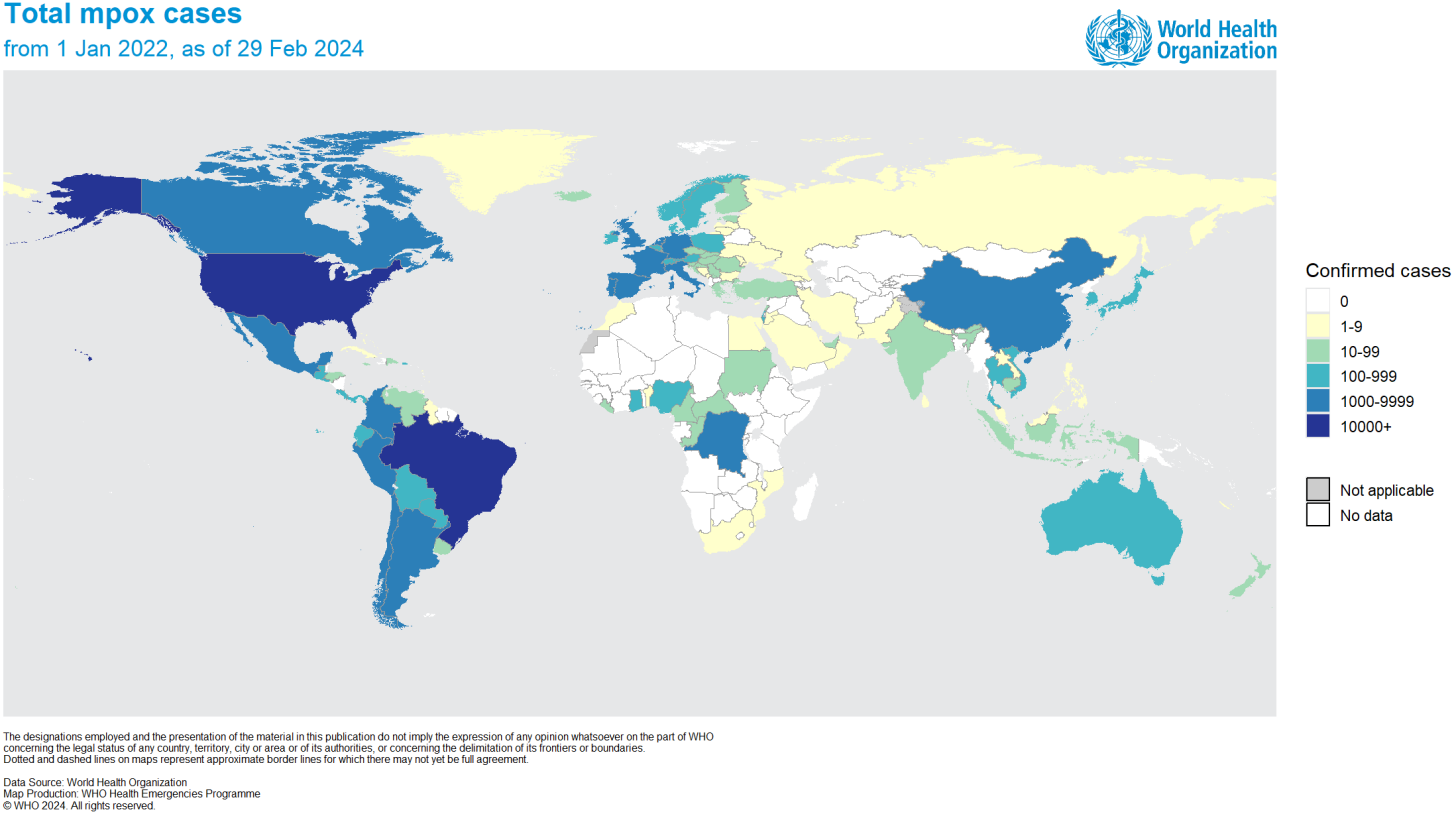
Distribution of confirmed MPXV cases worldwide as of February 29, 2024 (adapted with permission from World Health Organization, 2024).

The first case of mpox was found in a nine-month-old infant in 1970 in the Democratic Republic of Congo (DRC). Since 1970, there has been an increase in mpox outbreaks, mainly occurring on the African continent (Breman et al., 1980; Alakunle et al., 2020). Specifically, between 1970 and 1995, 388 out of 418 recorded cases of mpox were documented in Zaire (currently known as DRC) (Shchelkunov et al., 2001). Since May 2022, mpox cases have been reported in Europe and North America. As of February 29, 2024, the ongoing mpox outbreak has resulted in over 94,707 laboratory-confirmed cases, including 181 deaths. Approximately 715 cases have been reported worldwide monthly (World Health Organization, 2024). These cases are spread across 117 countries, of which only seven had previously reported cases of MPXV before 2022 (**Figure 1**). Due to the rising global cases, the World Health Organization (WHO) designated MPXV as a public health emergency of international concern (PHEIC) on July 23, 2022 (World Health Organization, 2022). According to the latest data received, the number of laboratory-confirmed cases reported monthly has increased by 1.6% compared to January, with the majority of cases originating from the USA (31.9%) and Europe (31.2%) (World Health Organization, 2024).

## Biological features

**Figure 2:**
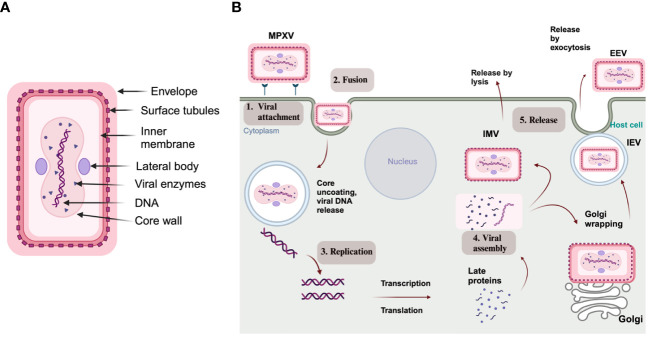
**(A)** Structure of MPXV; **(B)** Schematic representation of an MPXV replication cycle, including (1) viral attachment, (2) fusion, (3) replication, (4) viral assembly, and (5) release from the host cell (created via BioRender.com).

MPXV is characterized as an enveloped double-stranded (ds) DNA virus, and its genome size is approximately 197 kb, encoding nearly 190 proteins (Shchelkunov et al., 2002; Kumar et al., 2022). The virus structure includes a lipoprotein envelope, a viral core, and two lateral bodies, as depicted in **Figure 2A** (Witt et al., 2023). The genome comprises two variable regions on both the right and left sides and a conserved large central genomic region occupied with core genes. The variable regions are composed of genes responsible for encoding proteins related to virulence and determining host range, where significant differences between MPXV and VARV occur. Meanwhile, the core region encodes structural proteins and essential enzymes, which share 96.3% similarity with the core region of the VACV (Shchelkunov et al., 2001).

The ds nature of MPXV’s genetic material has advantages and disadvantages from a detection perspective. Unlike positive-sense single-stranded (ss) RNA viruses, like SARS-CoV-2, which can directly initiate the synthesis of viral proteins upon entering the host cell, DNA viruses need to first convert their DNA to RNA before expressing viral proteins (Durmuş and Ülgen, 2017). Consequently, MPXV can stay in the body longer before exhibiting noticeable symptoms in infected individuals (Bhalla and Payam, 2023). Hence, it can lead to the unnoticed spread of infection within the community. This phenomenon most likely played a role in the undetected spread of MPXV in various geographical areas, challenging researchers to develop new approaches, including diagnostic tools and biosensors. However, the advantage of DNA viruses is that they are comparatively more straightforward and more accurate in detecting using PCR tests than RNA viruses. DNA virus detection does not require reverse transcription before PCR (Bhalla and Payam, 2023).

Poxviruses, including MPXV, are recognized for their distinctive structure, which is typically brick-shaped or oval, with a size ranging from 200 to 250 nm (Cho and Wenner, 1973). Based on the genome sequence, MPXV is phylogenetically classified into two clades: clade 1, which is found in central Africa and the Congo basin, and clade 2, which is from West Africa. However, the phylogenomic analysis, including those from the 2022 outbreaks, revealed that these outbreaks were caused by a recently evolved clade called “hMPXV-1A” lineage B.1 (Luna et al., 2022).

## Pathogenesis

The virus can be transmitted from animals to humans or from humans to humans via direct and close contact, spreading through blood, body fluids, and dermal or mucosal injuries. During human-to-human transmission, MPXV enters through the upper respiratory tract, including the oropharynx and nasopharynx, or via intradermal routes (Li et al., 2022b). The clinical features of MPXV highly resemble smallpox disease, which also presents 10-14 days of incubation followed by two days of skin rash formation. Like smallpox, the development of rash includes phases such as macular, popular, vesicular, and pustular (Weaver and Isaacs, 2008; Hughes et al., 2014). Hence, the differentiation of these diseases by clinical presentation is quite challenging.

As with other poxviruses, MPXV replication occurs in the cytoplasm and undergoes through virally encoded RNA polymerase. As depicted in **Figure 2B**, MPXV pathogenesis includes viral particle attachment, fusion, viral genome replication, virion assembly, and release from the infected host cell. During these steps, two types of infectious forms of MPXV are produced: extracellular enveloped virus (EEV) and intracellular mature virus (IMV). The EEV is released through exocytosis and comprises a lipid membrane wrapped around the intracellular IMV particle originating from the Golgi apparatus or endosomes. On the other hand, the IMV is released during cell lysis and has a stable lipoprotein envelope, making it suitable for transmission between animals (Gong et al., 2022; Shi et al., 2022).

The entry fusion step involves a complex interaction with multiple receptors, namely heparan sulfate, glycosaminoglycans, and chondroitin (Montanuy et al., 2011; Hughes et al., 2014; Khanna et al., 2017). It has also been proposed that genes responsible for viral replication enzymes and structural proteins are highly homologous among OPXVs (Shi et al., 2022). Senkevich et al. (2005) proposed that the fusion entry mechanism is conserved among the poxvirus family, which suggests that this primary mechanism developed early in their evolution and remains unchanged. Hence, MPXV can possess standard features in its entry-fusion step with other members, especially VACV.

## MPXV proteins

The genes encoding the MPXV structural proteins are located within the highly conserved central genomic region and expressed in different forms of the mpox virus. The EEV form of MPXV expresses 25 membrane proteins, including C19L, A35R, and B6R (**Table 1**). In contrast, the IMV form expresses proteins such as A29, M1R, E8L, H3L, and L1R (Shchelkunov et al., 2002). According to Freyn et al. (2022), specific MPXV proteins, homologs of VACV, such as M1 and A29, are involved in the cellular entry process of mature virus (MV), and A35 and B6 are identified as contributors to the transmission mechanism on the enveloped virus (EV) surface. Among these proteins, A35 has been identified as an essential factor for poxvirus virulence. Studies have shown that the loss of A35 protein resulted in a 1000-fold attenuation in virulence (Freyn et al., 2022). Moreover, studies indicate that antibodies against the L1R protein, located in the outer membrane of MV, can prevent the virus from infecting cells, suggesting that L1R might also have a role in the viral entry step (Shi et al., 2022).

**Table 1:** MPXV proteins and their characteristics.

Location	Protein	Length (aa)	Protein sequence	Sequence alignment similarity (%) with VACV	Function	Cellular localization (when expressed in 293T cells)	Reference
IMV	A29L	110	1 mdgtlfpgdd dlaipateff stkaaknpet kreaivkayg ddneetlkqr ltnlekkitn 61 ittkfeqiek cckrndevlf rlenhaetlr aamislakki dvqtgrhpye	A27L 94.54%	viral replication, viral recognition, regulation of cell entry and viral egression	cytosol	Shchelkunov et al., 2002; Yong et al., 2020; Shi et al., 2022; Fang et al., 2023; Gao et al., 2023; Ye et al., 2023; Wang et al., 2023a.
	H3L	324	1 maaaktpviv vpvidrppse tfpnvhehin dqkfddvkdn evmqekrdvv ivnddpdhyk 61 dyvfiqwtgg nirdddkyth ffsgfcntmc teetkrniar hlalwdskff ielenknvey 121 vviiendnvi editflrpvl kaihdkkidi lqmreiitgn kvktelvidk dhaiftytgg 181 ydvslsayii rvttalnivd eiiksgglss gfyfeiarie nemkinrqim dnsakyvehd 241 prlvaehrfe tmkpnfwsri gtvaakrypg vmytfttpli sffglfdinv iglivilfim 301 fmlifnvksk llwfltgtfv tafi	H3L 93.52%	promotes binding to host cells and infectivity	–	Lin et al., 2000; Kugelman et al., 2014; Yefet et al., 2023.
	E8L	304	1 mpqqlspini etkkaisdar lktldihyne skpttiqntg klvrinfkgg yisggflpne 61 yvlstihiyw gkeddygsnh lidvykysge inlvhwnkkk yssyeeakkh ddgiiiiaif 121 lqvsdhknvy fqkivnqlds irsanmsapf dsvfyldnll pstldyftyl gttinhsada 181 awiifptpin ihsdqlskfr tllsssnheg kphyitenyr npyklnddtq vyysgeiira 241 attspvreny fmkwlsdlre acfsyyqkyi egnktfaiia ivfvfiltai lflmsqrysr 301 ekqn	D8L 97.74%	surface attachment and viral entry	endomembrane	Shchelkunov et al., 2002; Alkhalil et al., 2009; Yong et al., 2020; Fang et al., 2023.
	M1R	250	1 mgaaasiqtt vntlseriss kleqeanasa qtkcdieign fyirqnhgcn itvknmcsad 61 adaqldavls aatetysglt peqkayvpam ftaalniqts vntvvrdfen yvkqtcnssa 121 vvdnklkiqn viidecygap gsptnlefin tgsskgncai kalmqlttka ttqiaprqva 181 gtgvqfymiv igviilaalf myyakrmlft stndkiklil ankenvhwtt ymdtffrtsp 241 miiattdiqn	L1R 98.40%	viral particle assembling and entry	plasma membrane or inclusion body-like structures	Senkevich et al., 2002; Yong et al., 2020; Fang et al., 2023.
	L1R	152	1 mdhnqylltm ffadddsffk yfasqddess lsdilqitqy ldfllllliq sknkleavgh 61 cyeslseeyr qltkftdsqd fkklfnkvpi vtdgrvklnk gylfdfvisl mrfkkesala 121 ttaidpvryi dprrdiafsn vmdilksnkv ek		viral particle entry, viral infection neutralization, virion assembly	–	Su et al., 2005; Golden et al., 2008; Karki et al., 2018; Yong et al., 2020.
EEV	F13L (C19L)	372	1 mwpfvsvpag akcrlvetlp enmdfrsdhl ttfecfneii tlakkyiyia sfccnplstt 61 rgalifdklk evsekgikii vlldergkrn lgelqshspd infitvnidk knnvglllgc 121 fwvsddercy vgnasftggs ihtiktlgvy sdypplatdl rrrfdtfkaf nsaknswlnl 181 csaacclpvs tayhiknpig gvfftdspeh llgysrdldt dvvidklksa ktsidiehla 241 ivpttrvdgs syywpdiyns iieaainrgv kirllvgnwd kndvysmata rsldalcvqn 301 dlsvkvftiq nntkllivdd eyvhitsanf dgthyqnhgf vsfnsidkql vseakkifer 361 dwvsshsksl ki	F13 99.9%	viral packing and egression: formation of virus-specific wrapping complex; lipid metabolism	–	Schmutz et al., 1995; Smith and Vanderplasschen, 1998; Bárcena et al., 2000; Shchelkunov et al., 2001, 2002; Shiryaev et al., 2021; Patel et al., 2023; Srivastava et al., 2023.
	A35R	181	1 mmtpendeeq tsvfsatvyg dkiqgknkrk rviglciris mvisllsmit msaflivrln 61 qcmsankaai tdsavavaaa ssthrkvvss ttqydhkesc nglyyqgscy ilhsdyksfe 121 dakancaaes stlpnksdvl ttwlidyved twgsdgnpit kttsdyqdsd vsqevrkyfc 181 t	A33R 95.03%	formation of actin-containing microvilli and effective cell-to-cell spread of viral particles	plasma membrane	Roper et al., 1998; Perdiguero and Blasco, 2006; Yong et al., 2020; Fang et al., 2023; Wang et al., 2023a.
	B6R	317	1 mktisvvtll cvlpavvyst ctvptmnnak ltstetsfnd kqkvtftcds gyhsldpnav 61 cetdkwkyen pckkmctvsd yvselydkpl yevnstmtls cngetkyfrc eekngntswn 121 dtvtcpnaec qplqlehgsc qpvkekysfg eymtincdvg yevigvsyis ctanswnvip 181 scqqkcdips lsnglisgst fsiggvihls cksgftltgs psstcidgkw npilptcvrs 241 neefdpvddg pddetdlskl skdvvqyeqe iesleatyhi iimaltimgv iflisiivlv 301 cscdknndqy kfhkllp	B5R 96.53%	efficient spreading of infection, regulation of complement system of the host cell, contribution in wrapping steps of IMV to formulate EEV	perinuclear structures	Hooper et al., 2003; Bell et al., 2004; Fang et al., 2023; Tang et al., 2023; Wang et al., 2023a.
Other proteins	A42R	133	1 maewhkiied isknnkfeda aivdykttkn vlaaipnrtf akinpgevip litnhnilkp 61 ligqkfcivy tnslmdenty amelltgyap vspiviarth taliflmgkp ttsrrdvyrt 121 crdhatrvra tgn	A42R 98%	profilin-like, regulation and aggregation of the actin filaments	–	Shchelkunov et al., 2002; Kugelman et al., 2014; Minasov et al., 2022.

IMV, intracellular mature virus; EEV, extracellular envelope virus.

### IMV proteins

#### A29

MPXV A29, the ortholog of VACV A27, is a protein found on the viral envelope, particularly on IMV. It plays a crucial role in viral replication, the fusion of the virus with the host cell membrane, and viral egress (Shchelkunov et al., 2002; Gao et al., 2023). Additionally, MPXV A29 is the primary target in immunoassays that aim to detect MPXV (Shi et al., 2022). Shi et al. (2022) demonstrated the interaction of MPXV A29 protein with glycosaminoglycans (GAGs). They proposed a model for MPXV host entry, which includes (1) attachment of MPXV virion to the host cell surface through binding to heparan sulfate (HS), (2) initiation of fusion by host cell protease, and (3) eventual entry of virions into the host cell.

MPXV A29 shares a composition similarity of 94.54% with the VACV A27 (Wang et al., 2023a). Both proteins consist of 110 amino acids, which are categorized into functional parts, including an N-terminal signal peptide, a heparin-binding site (HBS), an α-helical coiled-coil domain, and a C-terminal anchoring domain (Vaázquez and Esteban, 1999). The HBS sequences of VACV 27A (STKAAKKPEAKR) and MPXV A29 (STKAAKNPETKR) differ in a single amino acid that occurred in a specific “KKPE” sequence that is essential for heparin-binding (Shih et al., 2009; Shi et al., 2022). However, despite this slight difference in the HBS sequence, Hughes et al. (2014) demonstrated that MPXV A29 and VACV 27A have a similar binding affinity to heparin.

#### H3L

The H3L antigen is expressed in the MV form, facilitating binding to host cells and enhancing infectivity (Lin et al., 2000). Additionally, it was discovered that anti-H3L antibodies can protect animals from a fatal attack (Davies et al., 2005). Moreover, H3L was identified as a target for T and B cells in vaccinated mice and humans (Davies et al., 2005). Specifically, H3L contains at least two recognized human leukocyte antigen (HLA) class I-restricted T-cell epitopes that can trigger a potent interferon (IFN) response. This makes H3L a focus of cellular immune responses (Ostrout et al., 2007).

Yefet et al. (2023) demonstrated that MPXV antigen H3L stimulates the production of antibodies and B cells in MPXV recoverees. They also reported that such individuals have a higher frequency of H3L-specific IgG+ B cells than those recently vaccinated against the virus (Yefet et al., 2023). Meanwhile, a report by Khlusevich et al. (2022) suggests that substituting residue 233A in H3L may disrupt a B-cell epitope, making it unrecognizable by anti-VACV polyclonal antibodies.

H3L is 93.52% homologous to VACV antigen H3L (Yefet et al., 2023). Yang et al. (2023a) chemically synthesized six MPXV protective antigens (PAs) and found that H3L had the lowest cross-reactivity compared to A29L, M1R, E8L, B6R, and A35R. Its cross-reactivity value against the VACV TianTan strain (VTT) - elicited anti-serum was 33%. They also reported that, upon examining the amino acid sequences of H3 and H3L, it was observed that the 233rd residue in H3L underwent a mutation from alanine to serine. This mutation likely contributed to the limited cross-reactivity of H3L against the anti-serum elicited by VTT. Mice vaccinated with recombinant H3L protein developed an elevated level of neutralizing antibodies (mean 50% plaque reduction neutralization test (PRNT50) 1:3,760) against VACV, allowing them to withstand intranasal exposures with fatal virus concentrations (Davies et al., 2005). As a result, the H3L protein is one of the main focus areas for MPXV vaccine development (Wang et al., 2023a).

#### E8L

E8L is an IMV surface membrane protein of 304 amino acids (Shchelkunov et al., 2002). According to UniProt, E8L (Q8V4Y0) is made up of three domains, namely the virion surface (1-275), transmembrane (276-294), and intra-virion domain (295-304) (Fantini et al., 2022). In a recent study by Fang et al. (2023), the E8L antigen was chosen for a polyvalent mRNA vaccine, and it was observed that its expression in 293T cells led to localization in the endomembrane. E8L works as a cell surface binding protein and specifically binds to chondroitin, regulating viral entry (Alkhalil et al., 2009).

It was previously shown that lipid rafts containing negatively charged gangliosides are one of the common entrance pathways OPXV uses (Byrd et al., 2013). Consequently, Fantini et al. (2022) identified the ganglioside-binding domain on the MPXV E8L protein. Using a multiparametric approach, they identified three linear epitopes overlapping with the annular ganglioside-binding motif of E8L, including amino acid sequences 43–62, 94–113, and 204–223. Consequently, these epitopes were suggested as immunogens in a vaccine formulation specific to the mpox.

#### M1R

M1R, an extensively preserved myristoylated surface membrane protein within IMV, plays a crucial role in the assembly and entry of viral particles (Tang et al., 2023; Zhang et al., 2023). As Yefet et al. (2023) highlighted, M1R is 98.4% homologous to the L1R of VACV. Meanwhile, Yang et al. (2023a) discovered that M1R has the most considerable cross-reactivity rate of 94% out of six MPXV PAs. This cross-reactivity may be due to B-cell epitopes, particularly those found in regions 69–91 aa and 137–155 aa regions, which overlap with those found in L1 (Heraud et al., 2006). Senkevich et al. (2002) mentioned that VACV L1R is found on the IMV membrane. In intracellular viruses, the outer domain of the L1R has three intramolecular disulfide bonds that face the cytoplasm.

M1R and L1R are essential targets for neutralizing antibodies in smallpox and cowpox viruses (CPXV) (Papukashvili et al., 2022). Fang et al. (2023) reported that M1R can be found in structures resembling the plasma membrane or inclusion bodies. Another study by Franceschi et al. (2015) demonstrated that M1R is essential for safeguarding mice against challenges, but its efficacy needs to be improved through Modified Vaccinia Ankara (MVA) vaccination. Despite their protective potential, booster vaccinations are necessary to ensure adequate protection (Franceschi et al., 2015).

#### L1R

L1R is a myristoylated membrane protein with a molecular weight ranging from 23 to 29 kDa, which shows significant conservation across OPXV (Su et al., 2005). It is situated on the surface of IMV and positioned beneath the envelope on EEV and can affect particle entry (Franke et al., 1990; Ravanello and Hruby, 1994; Wolffe et al., 1995). The general structure of L1R common to OPXVs comprises a cluster of α-helices that wrap a pair of two-stranded β-sheets linked by four loops (Su et al., 2005). Research suggests the L1R protein might play a role in the viral entry-fusion process. However, according to the surface plasmon resonance (SPR) analysis, the MPXV L1R exhibits notably low affinity for HS, resulting in an resonance unit (RU) value of -1.3, in contrast to the MPXV A29 protein, which showed an RU value of approximately 400. Similarly, the L1R’s affinity to other GAGs, such as DS, chondroitin sulfate A (CSA), and chondroitin sulfate E (CSE), was insignificant (Shi et al., 2022). Also, the L1R protein is in charge of virion assembly. Since it is present on the surface of IMV, it is also a potent subject for evoking neutralizing antibodies (Karki et al., 2018). Moreover, monkeys vaccinated with L1R exhibited low levels of MPXV-neutralizing antibodies at the challenge despite having elevated anti-L1R antibodies detected by immunogen-specific ELISAs. This suggests that the DNA vaccine-induced anti-L1R response may have included a significant proportion of non-neutralizing antibodies. Additionally, these sera demonstrated higher levels of VACV-neutralizing antibodies, suggesting that vaccination with the MPXV L1R ortholog might be advantageous for protecting against mpox (Hooper et al., 2004).

By incorporating the tissue plasminogen leader sequence (tPA) into L1R, Golden et al. (2008) generated neutralizing antibody responses, demonstrating a geometric mean titer (GMT) of 489. Notably, this robust neutralization response was achieved with just two vaccinations. In the study conducted by Hooper et al. (2004), they investigated the efficacy of the L1R DNA vaccine in generating IMV-neutralizing antibodies and providing safety. The DNA vaccines were governed using a gene gun, and the results revealed increased levels of L1R-specific antibodies in the sera of the two vaccinated monkeys after the booster shot. Consequently, gene gun vaccination with either the 4pox DNA vaccine or the L1R DNA vaccine induced a lasting memory response, persisting for at least one to two years (e.g., monkey L201-1) (Hooper et al., 2004). Hence, alleviating the illness could result from lowering the effective challenge dose through neutralizing the challenging virus.

### EEV proteins

 

#### F13

F13 (P37) is a critical envelope protein that is comprised of 372 amino acids. The MPXV gene C19L encodes it and is also called F13 (Patel et al., 2023). The F13 protein is located on the inner surface of the EEV outer membrane (Schmutz et al., 1995; Smith and Vanderplasschen, 1998). It is responsible for viral packaging and release from the host cell, facilitating viral spread and multiplication (Srivastava et al., 2023). Additionally, studies have shown that the P37 protein interacts with host membrane proteins Rab9 and TIP47 to create a virus-specific wrapping complex that is essential for the enveloped virus (Shiryaev et al., 2021; Srivastava et al., 2023). In addition to its primary function, the F13 protein also serves as an enzyme and participates in lipid metabolism (Bárcena et al., 2000).

**Figure 3:**
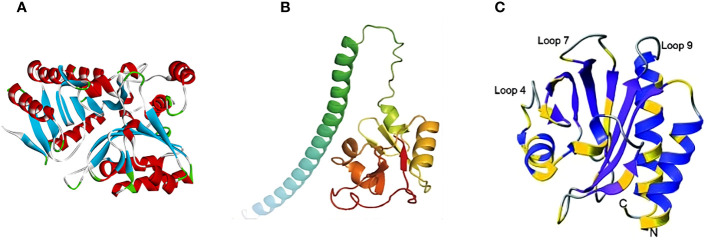
Predicted 3D structures of MPXV proteins: **(A)** F13 (adapted with permission from Patel et al., 2023); **(B)** A35R (adapted with permission from Zheng et al., 2022); and **(C)** A42R (adapted with permission from Minasov et al., 2022).

Several studies emphasized the importance of the P37 protein in the MPXV virus as a potential target for FDA-approved drugs. Shiryaev et al. (2021) noted that it has some significant benefits for virtual screening, like its relatively small size and the fact that homologs are absent in humans. The study performed high-throughput virtual screening (HTVS) to identify FDA-approved drugs with higher binding affinity against MPXV. Similarly, Patel et al. (2023) predicted the 3D structure of the F13 envelope protein using Alphafold (**Figure 3A**), built a homology model, and evaluated it using docking, binding pose metadynamics, and molecular dynamics (MD). Likewise, another study found 15 multitargeting FDA-approved drugs that may inhibit P37 (viral packing and release), topoisomerase 1 (viral DNA replication and transcription), and thymidylate kinase (viral DNA synthesis) (Srivastava et al., 2023).

Li et al. (2022a) found that the F13 sequence is highly conserved in MPXV and VARV, and similarity ranges from 97.58% to 99.73%. Similarly, Srivastava et al. (2023) conducted a protein-protein blast analysis, revealing a sequence alignment similarity of 99.9% between MPXV and VACV. As a result, tecovirimat, formerly approved for treating smallpox, has shown potential for treating mpox. They identified the structure of the MPXV F13 protein and its residues interacting with tecovirimat through molecular simulations. Furthermore, MD analysis confirmed the drug’s efficacy against mpox (Li et al., 2022a). Additionally, there are recent studies that provide an overview of potential antivirals against P37 (Wang et al., 2023b; Ashley et al., 2024) and other mpox proteins (Bajrai et al., 2022; Kaur et al., 2023).

#### A35R

A35R, a homolog of VACV A33R, is an envelope glycoprotein of EV, contributing to the formation of actin-containing microvilli and facilitating the effective cell-to-cell spread of viral particles (Roper et al., 1998; Perdiguero and Blasco, 2006). According to Wang et al. (2023a), the A35R protein shares 95.03% similarity with the A33R protein from VACV. Su et al. (2010) found that VACV A33R is a homodimeric transmembrane protein that undergoes O- and N-glycosylation at the N-125 and N-135 sites, while MPXV A35R does not possess the corresponding N-125 site. Moreover, it was shown that a few amino acid differences between these two proteins could influence the effectiveness of the smallpox vaccine in providing cross-protection against mpox. For instance, the anti-A33 monoclonal antibodies (mAbs) 1G10 and 10F10 showed high specificity for VACV A33R. However, in the case of MPXV A35R, 10F10 demonstrated an affinity to MPXV A35R, while 1G10 resulted in no binding (Golden and Hooper, 2008). The 3D structure of MPXV A35R was predicted by Zheng et al. (2022) using AlphaFold2 (**Figure 3B**). Consequently, the study showed the structural similarity of MPXV A35R and VACV A33R, especially in their globular domains (Zheng et al., 2022).

Fang et al. (2023) generated a polyvalent mRNA vaccine candidate, MPXVac-097, using five MPXV antigens such as A35R, B6R, A29L, E8L, and M1R linked by tandem dimer peptide linkage. As a result, after the second and third doses of the MPXVac-097 vaccination, antibody titer levels of A35R and E8L increased significantly, indicating a robust antibody response. However, the response was moderate to M1R and low to A29L and B6R antigens. The localization of the A35R protein to the plasma membrane was identified after expressing the A35R antigen in 293T cells (Fang et al., 2023). Three anti-MPXV A35 nanobodies from a non-immunized alpaca heavy-chain antibody (VHH) library were recently designed (Meng et al., 2023). As a result, VHH-1 demonstrated the highest affinity and specificity against MPXV A35R, with a half-maximal effective concentration EC_50_ of 0.010 µg/mL. Similarly, as the result of analysis using the protein A biosensor, VHH-1 demonstrated binding to the mpox A35R protein with an affinity constant of 54 nM, determined via the biolayer interferometry (BLI) assay, thereby providing a fundamental basis for the potential advancement of the diagnostic instruments for the mpox virus (Meng et al., 2023).

#### B6R

B6R is a glycoprotein that undergoes reversible lipid modifications called palmitoylation (Tang et al., 2023). These modifications are considered a crucial mechanism of protein trafficking to the membrane. Hence, it is essential for localizing B6R on the surface membrane of infected cells and the EEV (Guan and Fierke, 2011; Tang et al., 2023). B6R is the homolog of VACV B5R, showing a similarity of 96.53% (Wang et al., 2023a). Like B5R, B6R is vital for the efficient spreading of infection and is involved in the regulation of the complement system of the host cell (Smith and Vanderplasschen, 1998; Tang et al., 2023). B5R also contributes to formulating EEV during the wrapping steps of IMV (Bell et al., 2004). Fang et al. (2023) selected the MPXV B6R antigen as one of the candidates for inclusion in a polyvalent mRNA vaccine candidate, MPXVas-097. As discussed earlier, they showed a low antibody titer after a three-dose vaccination. The same study identified that its expression in 293T cells led to localization in perinuclear structures. Moreover, the B6R protein was recognized as the primary target for rt-PCR assay targeting MPXV. Li et al. (2006) designed an rt-PCR assay, namely a B6R assay, specifically detecting B6R envelope protein. As a result, all 15 strains of MPXV were detected at 10 ng with the B6R assay, and no cross-reaction was observed with other OPXV and bacterial strains.

### Profilin-like proteins

#### A42R

The A42R protein is encoded by the gp153 locus of the MPXV virus, and its amino acid sequence highly resembles profilin proteins (Minasov et al., 2022). Profilins are actin-binding proteins that regulate and aggregate actin filaments (Pinto-Costa and Sousa, 2020). It was found that VACV A42R, homolog to profilin, shares 98% similarity with MPXV virus A42R, and it is a late synthesized protein that exhibits a weak affinity for actin (Minasov et al., 2022). In the previous studies, knockout of the open reading frames (ORF) for VACV A42R and CPXV A42R showed that they are not crucial for poxvirus replication *in vivo* (Blasco et al., 1991). However, according to the authors, they may play a vital role during viral replication in various cell lines (Minasov et al., 2022).

Minasov et al. (2022) identified the structure of MPXV A42R protein (**Figure 3C**) through the single-wavelength anomalous dispersion (SAD) technique using X-ray diffraction data collected from crystals of seleno-methionine derivatized protein at a resolution of 1.52 Å. The principle of X-ray diffraction lies in the interaction of X-rays with the electron clouds on the crystal atoms, creating the diffraction pattern (Liebschner et al., 2019). The protein has an asymmetric structure composed of two polypeptide chains, chains A and B. Chain A is a full-length chain containing 133 amino acid residues. It begins with N-terminus alanine, which originates from the tobacco mosaic virus protease. On the other hand, chain B lacks N-terminal alanine and starts from the second amino acid up to the 133rd. Generally, the structure contains a seven-stranded antiparallel beta-sheet encircled by three alpha helices and a partially formed helix (Minasov et al., 2022). Structural analysis of MPXV A42R with bovine and human profilin proteins uncovered notable differences in critical functional regions. Specifically, it was revealed that, unlike profilins, MPXV A42R has a weak affinity for actin and no affinity for poly (l-proline). In addition to this, A42R may establish specific interactions with phosphatidylinositol lipids. This suggests that understanding the function of cellular profilin may not be sufficient to predict the role of MPXV A42R (Minasov et al., 2022).

## Rapid detection techniques

Several detection techniques for mpox are being used nowadays. For instance, lateral flow assay (LFA), also known as immunochromatography, is driven by capillary action and provides quick detection employing colloidal gold nanoparticles as immunolabels, producing results in 10-15 minutes at a cheap cost and with simple standardization (Urusov et al., 2019; Wang et al., 2022; Ye et al., 2023). Immunology-based LFA assays are popular for quick test times and convenience (Qriouet et al., 2021). Ye et al. (2023) developed a colloidal gold immunochromatographic method for monkeypox virus detection that uses the A29 17-49 peptide sequence as the immunogen and produces monkeypox-specific mAbs. Rapid test strips were developed using the double-antibody sandwich approach, which has great specificity and sensitivity. It was found that two specific antibodies, namely mAb-7C5 and 5D8, resulted in the best sensitivity and detection of the limit of 50 pg mL^−1^ for the A29 protein (**Table 2**). Moreover, the test strips did not show cross-reactivity with other OPXVs, including VACV and CPXV, and other infections, such as SARS-CoV-2 and influenza A and B (Ye et al., 2023).

**Table 2:** Current approaches for rapid MPXV protein detection.

Method	Description	Target protein	Detection time	Detection limit	Reference
LFA	Immunochromotographic assay based on colloidal gold nanoparticles on the double-antibody sandwich principle for detection of mpox	A29	15 min	50.0 pg/mL	Ye et al., 2023
LFIA	Colorimetric-fluorescent dual-signal nanotag-based LFIA sensor	A29L	15 min	0.5 and 0.0021 ng/mL	Yang et al., 2023b
SPR	Immediate and non-labeled assessment of affinity and binding kinetics in real-time	A29	-	*K*_D_ for HS 2.6 × 10^−7^ M; DS 6.2 × 10^−7^ M; CSA 8.4 × 10^−7^ M; CSE 3.1 × 10^−7^ M	Shi et al., 2022
		C19L	-	*K*_D_ between synthetic antibody 62 and C19L protein was 0.1 nM, and WT C19L antibody and C19L protein was 0.8 nM	Shabani et al., 2023
Label-free SERS	Enhanced precision and sensitivity, along with quicker outcomes, achieved by integrating molecular data with the plasmonic characteristics of metallic nanostructures	A29L, M1R, B6R, and A35R in the serum	5 min	5 ng/mL (A29L)	Zhang et al., 2023
EIS	Paper-based highly porous AuNS-treated LSG electrochemical sensor	A29L	15 min	3.0 × 10^–16^ g/mL	de Lima et al., 2023

LFA, Lateral Flow Assay; SPR, Surface Plasmon Resonance; *K*_D_, dissociation constant; HS, heparan sulfate; DS, dermatan sulfate; CSA, chondroitin sulfate A; CSE, chondroitin sulfate E; SERS, Surface-Enhanced Raman Spectroscopy; LFIA, Lateral Flow Immunoassay; EIS, electrochemical impedance spectroscopy; AuNS, gold nanostructures; LSG, laser-scribed graphene.

Yang et al. (2023b) established a dual-signal nanotag-based lateral flow immunoassay (LFIA) system for swift and sensitive detection of the A29L protein. They investigated the ideal reaction time for LFIA, examining the T-line’s signal-to-noise ratio (SNR) across various time intervals. The findings indicated that a reaction period of 15 minutes proved adequate for quantitatively detecting MPXV. The dual-signal SiO_2_-Au core dual-QD shell (DQD) nanocomposite (Si-Au/DQD)-based LFIA has a colorimetric sensitivity of 0.5 ng/mL and fluorescence sensitivity of 0.021 ng/mL, outperforming the standard AuNP-based LFIA and ELISA procedures by 238 and 3.3 times, respectively (Yang et al., 2023b).

Virus proteins, including mpox, were also analyzed using alternative techniques such as SPR. Shi et al. (2022) showed the interaction of MPXV A29 protein with GAGs, HS, dermatan (DS), CSA, and CSE using SPR. As a result, the obtained dissociation constant (*K*_D_) values (**Table 2**) indicated that MPXV A29 exhibited a strong affinity for GAGs, including HS, DS, and CS. Likewise, the same study employed SPR to study the affinity of the MPXV L1R protein to GAG. Shabani et al. (2023) designed a new synthetic anti-MPXV C19L antibody (antibody 62) based on a heavy chain of human antibodies and a small peptide fragment at its beginning. The docking and molecular simulation analysis of the designed anti-MPXV C19L antibodies were applied to select the one with superior stability. The interaction between the C19L protein and the synthetic and wild-type anti-C19L antibodies was analyzed using SPR. The findings showed that synthetic antibodies’ *K*_D_ value was lower than wild antibodies, 0.1 and 0.8 nM, respectively, meaning that synthetic antibodies had a higher affinity for MPXV C19L protein (Shabani et al., 2023). In this manner, SPR enabled a label-free, direct, and real-time quantitative assessment of molecular interactions (Shi et al., 2022).

Because of its low cost, speed, high sensitivity, specificity, and noninvasiveness, surface-enhanced Raman spectroscopy (SERS) has been frequently utilized to detect harmful microorganisms (Huang et al., 2021). Unlabeled detection eliminates the need for attaching molecular markers like specific antibodies and nucleic acid sequences onto nanoparticle surfaces, simplifying the preparation of the enhanced substrate and allowing for rapid sample detection (Zhang et al., 2023). Zhang et al. (2023) also emphasized that despite SERS’s robust detection features, some technical hurdles in unlabeled virus identification must be overcome, such as the difficulty in recording signals and insufficient applicability because of the size distinction between SERS “hot spots” and viruses. Consequently, they used silver nanoparticles incubated with iodine ions and aggregated with calcium ions as substrates to resolve the limitation of unlabeled MPXV detection of the SERS. As a result, the new approach showed the detection of MPXV A29L protein at a concentration as low as 5 ng/mL and MPXV DNA at levels as low as 100 copies/mL within 2 minutes, which is close to the lower limit of PCR detection but faster. Furthermore, SERS has shown excellent potential for quantitatively detecting MPXV as it was able to identify four distinct MPXV proteins, including A29L, M1R, B6R, and A35R, in the serum in 5 minutes (Zhang et al., 2023).

**Figure 4:**
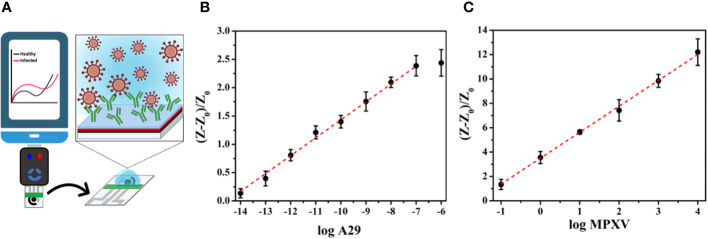
**(A)** Schematic illustration of an LSG nanobiosensor-based electrochemical assay for MPXV detection linked to a portable potentiostat controlled via a smartphone; **(B)** The analytical curve presenting normalized resistance to charge transfer (*R*_CT_) values against the logarithm of the A29 protein concentration; **(C)** The analytical curve presenting the normalized *R*_CT_ values against the logarithm of the titered MPXV sample (adapted with permission from de Lima et al., 2023).

Electrochemical biosensors are another attractive approach that offer typical rapid testing times, convenient device portability, and compact device size, which lower sample volumes required for testing and remove sample pretreatment steps, making them suitable for point-of-care applications (POC). To ensure optimal use in POC settings, electrochemical devices must be reproducible and easily accessible, allowing them to be disposable and removing the requirement for trained personnel (Torres et al., 2021). de Lima et al. (2023) reported the first electrochemical POC assay for MPXV protein detection using a paper-based laser-scribed graphene (LSG) nanobiosensor (**Figure 4A**). The results showed that the test required a small amount of sample (2.5 µL), detection within 15 min, and a limit of detections (LODs) of 3.0 × 10^–16^ g mL^–1^ for A29 protein and 7.8 × 10^–3^ PFU L^–1^ for titered MPXV. The analytical curves for A29 protein and MPXV viral loads at various concentrations showed a linear correlation with a determination coefficient (*R* ^2^) of 0.998 and 0.996, respectively (**Figures 4B, C**). Additionally, no instances of cross-reaction were detected when the nanobiosensor was tested alongside other poxvirus and non-poxvirus strains (de Lima et al., 2023).

## Conclusion and future directions

Mpox, a zoonotic disease caused by MPXV, presents challenges in diagnosis due to symptom similarities with other infectious diseases. Despite the current decrease in confirmed infection cases, addressing the ongoing threat of mpox remains imperative, especially considering its potential emergence in previously non-endemic areas. Therefore, rapid and specific detection methods are crucial for controlling its global spread. Consequently, this review provided a comprehensive overview of the characterization of mpox proteins and the development of rapid techniques for MPXV detection. Specifically, we highlighted pathogenesis, the roles and structural characteristics of EEV (C19L, A35R, and B6R), IMV (A29, M1R, E8L, H3L, and L1R), and profilin-like proteins. Additionally, recent studies in diagnostic techniques such as LFA, LFIA, SPR, SERS, and EIS using these mpox proteins demonstrated promising paths for rapid detection of MPXV. Recently, viral detection methods based on aptamers have been developed with considerable success (Xi et al., 2021; Shola David and Kanayeva, 2022). Unlike protein biorecognition elements, including antibodies, nucleic acid aptamers can be readily paired with various electrochemical and optical sensing methods, opening new avenues for sensitive and rapid detection (Zhang et al., 2023). However, it is crucial to note that despite the advancements, there is still a gap in understanding most mpox proteins. Notably, while the 3D structure of A42R has been revealed, predicted 3D structures are available for proteins C19L and A35R, while others still need to be explored. This emphasizes the necessity for further research on mpox protein structure and function. Enhancing knowledge of mpox proteins is essential for improving diagnostic techniques to address emerging viral infections effectively.

## Author contributions

DK: Conceptualization, Funding acquisition, Project administration, Supervision, Visualization, Writing – original draft, Writing – review & editing. KS: Data curation, Visualization, Writing – original draft. AB: Data curation, Visualization, Writing – original draft.
